# Evaluation of patients´ perspective on a multimorbidity patient-centered care model piloted in the chilean public health system

**DOI:** 10.1186/s12889-023-17220-3

**Published:** 2023-11-16

**Authors:** Jaime C. Sapag, Mayra Martínez, Paula Zamorano, Teresita Varela, Álvaro Téllez, Esteban Irazoqui, Paulina Muñoz

**Affiliations:** 1 Department of Family Medicine, Pontificia Universidad Católica de Chile, Santiago, Chile; 2 Department of Public Health Pontificia, Universidad Católica de Chile, Santiago, Chile; 3 Dalla Lana School of Public Health, University of Toronto, Toronto, Canada; 4 Facultad de Medicina, Innovación ANCORA UC, Pontificia Universidad Católica de Chile, Santiago, Chile; 5 Departamento de Ciencias de la Salud, Pontificia Universidad Católica de Chile, Santiago, Chile; 6 Diagonal Paraguay, Santiago, 362 Chile

**Keywords:** Multimorbidity, Patient, Perspective, High-risk, Implementation

## Abstract

**Background:**

The progressive multimorbidity explosion has challenged Chile’s health systems and worldwide. The Centro de Innovación en Salud ANCORA UC implemented a new Multimorbidity Patient-Centered Care Model in Chile.

**Objective:**

Evaluate the perspective of high-risk patients about the core elements of the model.

**Methodology:**

We conducted a cross sectional telephone-based survey that considered the application of a 13 items questionnaire. Of them, nine were Likert scale questions with scores from 1 to 7, one dichotomic question, and three open-ended questions. 231 high-risk patients who received care through the model at primary care centers participated in the study. Quantitative data were encoded, consolidated, and analyzed with the SPSS software. We performed descriptive and analytic statistics techniques to assess different variables and their potential associations. Thematic analysis was conducted for qualitative data.

**Results:**

The overall score was 5.84 (range: 1 to 7), with a standard deviation of 1.25. Questions with the best scores were those related with personalized care and the primary care teams. The lowest scored was for the item regarding the continuity of care between primary nurses and inpatient care at the hospital. There was a difference in patient outcomes depending on their health center. Regarding sociodemographic characteristics, age did not significantly affect the results.

**Conclusions:**

The study reveals the perceptions about a complex multimorbidity intervention from the patient’s perspective. It complements the impact on health services utilization evaluation that supports decision-makers currently scaling up a similar strategy in our country and could be considered in other countries dealing with non-communicable diseases.

**Supplementary Information:**

The online version contains supplementary material available at 10.1186/s12889-023-17220-3.

## Introduction

Multimorbidity defined as two or more chronic conditions [1], is challenging for health systems, patients, and their families [2, 3]. The high burden of disease, the elevated consumption of health services, and the impact on a poorer quality of life must be addressed urgently [4, 5]. More than 9 million people in Chile have multimorbidity, and more than 11 million have at least one non-communicable chronic disease [6]. Although strategies have been implemented to address this matter, when comparing data from the National Health Survey of 2010 and 2017, negative outcomes have increased, and the problem is even more evident [7–9].

Chile’s primary health care system is based on a single diagnosis approach organized with health goals and key performance indicators that entail significant fragmentation and constraints for health care innovations [10–12]. Despite the Family and Community Health care model was implemented in primary care more than two decades ago, chronic disease health services still have a focus on diagnostic approach fragmenting primary care services [13, 14]. Even more, coordination and continuity of care between primary, secondary, and tertiary care directly impact patients, their families, and the health system as they frequently use health services due to unprevented complications [15, 16].

Consequently, in Chile, patients perceive challenges in terms of quality and access, lacking continuity of care from their primary care team, excess medications, and increasing costs [17, 18]. Also, they do not find space and time to express their real needs and preferences [9, 19]. Even more, the continuous lack of information for correct decision-making ultimately results in an unattended need, thus creating a flawed health system [20]. Therefore, measuring patients’ experiences and perspectives can add value in assessing the quality of care and in the design and continuous improvement of diverse healthcare setting [21]. For example, dimensions like communication, access, patient education, and discharge process have been evaluated for planning and evaluating health care delivery [22].

In response, the Centro de Innovación en Salud ANCORA UC (CISAUC), in conjunction with the Servicio de Salud Metropolitano Sur Oriente (SSMSO) and the National Health Fund (FONASA), piloted a new model of care for people with multimorbidity: the Multimorbidity Patient-Centered Care Model (MPCM) [23]. The objective was to implement a new care model that could change the single-diagnosis care approach towards care based on patients’ needs through a comprehensive care multimorbidity approach [23]. This model highlights fundamental elements of the Family and Community Health Model already installed in Chilean primary health care and adds case management, self-management, and risk stratification. This way, clinical intervention strategies and new roles were designed according to person’s risk. This model was implemented in seven primary care centers and three hospitals of high complexity at the SSMSO between 2017 and 2020. The contribution of MPCM has demonstrated a positive impact on health services utilization, risk of death, and the management of health resources [24].

The MPCM implementation process was in three phases. During the first phase, activities were carried out to prepare the teams through training instances, dissemination and communication of the model, and preparation of the minimum conditions for implementation. Second, clinical activities began with the permanent field advice of the CISAUC expert team seeking to mitigate or address barriers to secure the implementation of the proposed intervention. Third, an impact assessment was carried out on health services utilization and qualitative evaluation of health teams and patients. Since patients at the highest risk have a higher disease burden, consume more resources, and therefore use the health system more frequently, it is relevant to evaluate the perspective of patients who received care with the new model of care.

The MCMP core elements are risk stratification, case management, self-management, shared responsibility, and continuity of care, where each intervention strategy was designed and implemented in the real context. Therefore, generating knowledge on the impact of each element on patient satisfaction through questions regarding each core element of the MPCM could add value for further complex interventions in the field. The present study aims to evaluate the perspective of high-risk patients about the core elements of MPCM.

## Methodology

The study used a cross-sectional telephone-based survey (supplementary material), given that data collection was done during the COVID-19 pandemic. A total of thirteen questions were considered to obtain the high-risk patients’ perspectives about the central elements of the MPCM implemented in seven primary care centers (PHC). The PHC are located in the southeast of Santiago, Chile, and belongs to the municipalities of La Florida, Puente Alto, and La Pintana. The population covered by each PHC ranged from 22,000 to 35,000 persons.

The total universe of high-risk patients was 692, where 67.5% were female, average number of non-communicable chronic diseases was 9.1, and their average age was 70.2 years. The sample size was calculated for a minimum of 231 patients considering a standard error of 5% and a confidence interval of 95%.

One of the seven PHC that participated in the MPCM was not included in the scope of this study because of a delay in its implementation process. The application of the survey was carried out between March and July 2021. The selection criteria were based on the patient receiving intervention under this new model in 2020. Subsequently, those patients who received at least one face-to-face consultant during 2020 were selected. As participants received care in one of the six participant PHCs, a stratified random sampling approach was considered, with proportional groups, being PHCs the strata. The same PHC teams were asked to provide contact information for the patients. Then, the sample was created and the calls were initiated.

We found no specific instrument to measure the researchers’ interest in this study. The research team designed the questions, taking as a reference the theoretical-conceptual definition of the core elements of the MPCM mentioned above and described in related publications [24, 25]. Subsequently, the survey as a draft was shared with the CISAUC expert group for review. Then, the questions were consolidated by the research team.

**Fig. 1:**
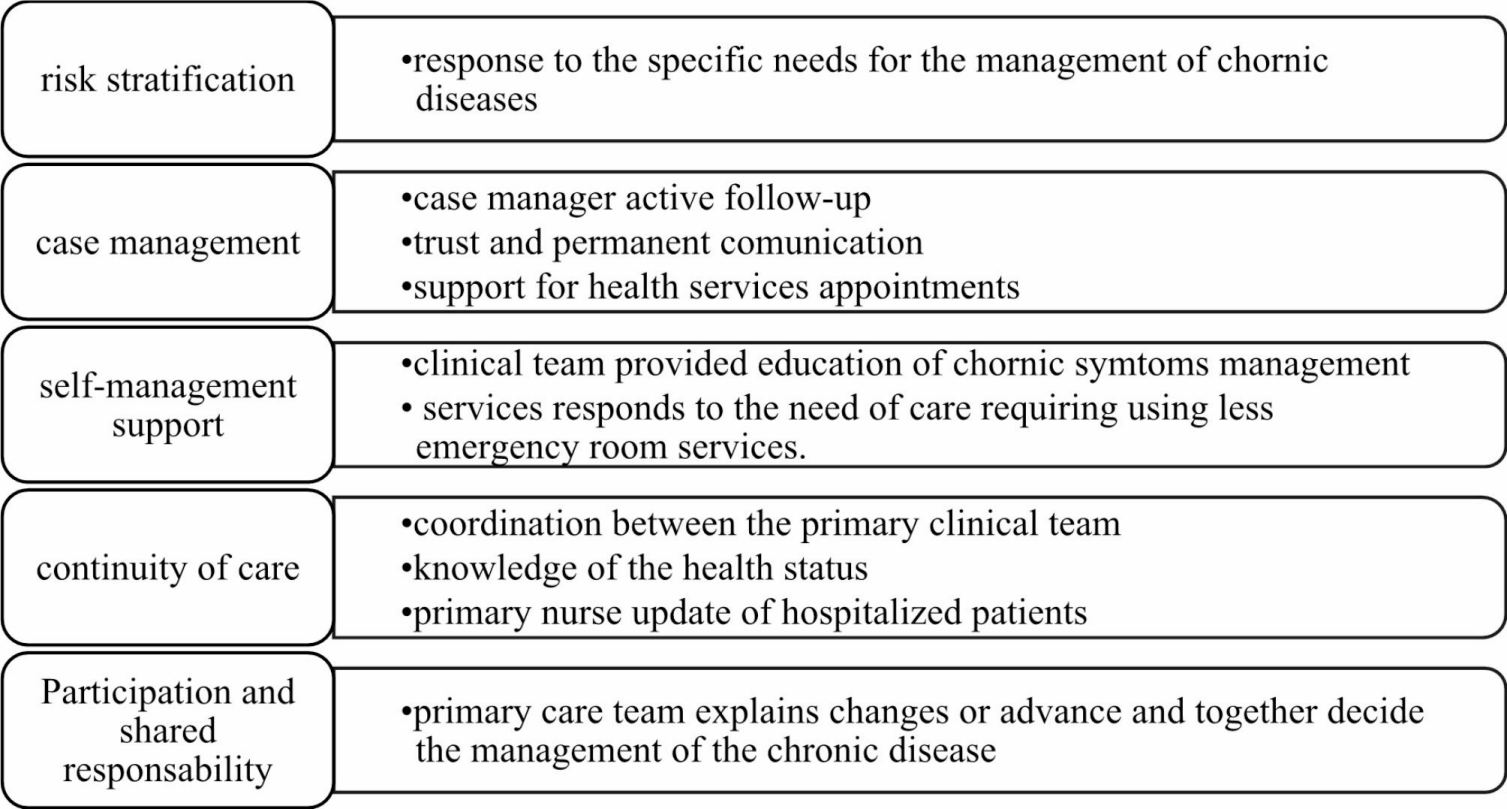
MPCM core elements and the activities evaluated in the survey

The survey had ten quantitative questions using a Likert Scale from one to seven (considering that the school evaluation scale in Chile is from 1 to 7), one dichotomic question with an open-ended qualitative component, and two qualitative using open-ended questions. Questions one to nine seek to inquire about assessing the core elements of MPCM through clinical activity, as shown in Fig. 1 (risk stratification, continuity of care, self-management support, and participation and shared responsibility). Question ten asked about overall satisfaction with the care received for the treatment of chronic illnesses. Question eleven was a dichotomic one (yes/no) focused on perception of service improvement, but with an open-ended qualitative component to comment on specific aspects. Questions twelve and thirteen were qualitative and asked about the most valued aspects, and areas for improvement.

### Data collection

Data collection was carried out by three professionals from the social sciences field who were research assistants. First, they received a four-hour training on the research, the survey, its application, and the ethical processes that should be carried out with each patient. Then, each professional was assigned to a database with the list and contacts of patients. The survey application was initiated by telephone according to a protocol developed for the study. If the patient could not respond to the survey, it was offered to schedule a new call. If the patients could not be reached after four attempts, the professionals called the next person on the list. It is estimated that 20% of patients could not be contacted and less than 5% of those who managed to be contacted refused to answer the survey. The application of the telephone survey lasted between 20 and 40 min, depending on the patients’ reality. An informed consent process – which took about other five minutes - was carried out before starting the questionnaire. Most of patients responded the survey without the support of somebody else. There were just 15 cases were family members/significant others played a role in the process, either responding on their behalf [13] or supporting the participant [2] to respond the survey.

The results were recorded in the SurveyMonkey platform at the time of the survey application.

### Data analysis

Data were extracted from the SurveyMonkey platform and analyzed in the SPSS version 17 statistical program. The analysis of questions one to nine was performed by calculating each core element’s median, mean, and standard deviation. Questions were analyzed independently of age and gender, and an overall score was calculated for the nine questions.

The quantitative components of question 11 was analyzed based on the response rate of yes/no answers. The qualitative component of questions 11 (those who identified improvements indeed), as well as questions 12 and 13, were assessed using a thematic analysis process [26, 27]. First, all transcripts were revised, and main key concepts were identified in the full data set in order to define preliminary codes. Then an in-depth exploration to identify and agree in terms of subcodes was done. Data were organized in categories following the main theme of each question (1. concrete perceived improvements in the provision of care with the new model -in case they noticed improvements-; 2. the most valued aspects of the model; and 3. the least valued aspects - areas for improvement -)., as well as considering the core elements of the MPCM, and potential emerging topics. Then, main categories were analyzed to identify specific dimensions within them based on how frequent they were mentioned as well as their relevance for the study focus of interest. Special attention was paid to coding accuracy and inter-coders’ reliability during the analysis process. Triangulation techniques (methodological, data source, researchers and theoretical) were also applied. Data saturation was evaluated, both operationally and theoretically. The analysis categories included all the core elements mentioned in the MPCM except for risk stratification. It was excluded because only high-risk patients were surveyed. Other emergent themes were also considered in the analysis, for example, general services characteristics.

Finally, a wider focus on mixed methods considerations [28] was helpful to identify the strongest findings of this study and to deep the discussion and conclusions considering both quantitative and qualitative data.

## Results

The results are presented organized in three sections: quantitative (questions one to ten), qualitative dichotomic (question eleven), and open-ended qualitative questions (twelve and thirteen).

The mean age of the participants was 66 years, with a standard deviation of 11.9 years. The distribution of sex was 74% women and 26% men.

### Quantitative results

Chronic disease characterization, sex, mean number of non-communicable diseases (NCD) and mean number of medications, and ACG resource utilization band were analyzed and described on the surveyed patients as shown in Table 1. Hypertension, dyslipidemia, and diabetes condition were the most frequent. The less frequent were bipolar disorder and osteoporosis. Most of the patients were female and had an average of 8 NCD and 14 drugs.

**Table 1:** Characterization of the sample of participants

Non communicable chronic disease	Frequency
Hypertension	93,75%
Dyslipidemia	82,39%
Diabetes	69,89%
Persistent asthma	36,36%
Congestive heart failure	35,23%
Chronic obstructive pulmonary disease	23,86%
Ischemic heart disease	21,02%
Low back pain	13,07%
Chronic kidney failure	12,50%
Rheumatoid arthritis	4,55%
Osteoporosis	3,98%
Bipolar disorder	1,14%
Resource Utilization band 4 high risk patients	60,23%
Resource Utilization band 5 high risk patients	39,77%
Gender	71% F / 29% M
NCD average	8,85 (min 2- max 21)
Average of drugs per patients	14,6

The overall score mean of questions one to nine based on the 231 valid cases of this section of the survey was 5.84 on a scale of 1 to 7 (minimum 1.22 - maximum 7; standard deviation 1.25). There were not statistically significant differences by age or gender. Regarding the MPCM core elements, the results indicated that risk stratification was the element that had the best score (6.08), followed by case management trust and permanent communication (score: 6.06). On the contrary, continuity of care (primary nurse update of hospitalized patients) had the lowest score (5.45), together with self-management support (services response to the need of care requiring using less emergency room services) (score of 5.74), as shown in Table 2.

**Table 2:** Core elements results

MPCM Element	Clinical Activity	Median	Standard deviation
Risk stratification	response to the specific needs for the management of chronic diseases	6.08	1.4
Case management	case manager active follow-up	5.85	1.68
	trust and permanent communication	6.06	1.6
	support for health services appointments	5.82	1.8
Self-management support	clinical team provided education of chronic symptoms management	5.78	1.75
	services response to the need of care requiring using less emergency room services	5.74	1.79
Continuity of care	primary care team is coordinated and up to date with my health condition	5.97	1.54
	primary nurse update of hospitalized patients	5.45	2.09
Participation and shared responsibility	primary care team explains changes or advance and together with the patient decide the management of the chronic disease	5.84	1.87

*Values of Likert Scale 1 to 7.

In terms of question 10, which asked about overall satisfaction “with the care you receive for the treatment of your chronic illnesses”, the overall score mean was 5.84 on a scale of 1 to 7 (with a 58.9% of users indicating the maximum score: 7).

Question eleven focused on the perception improvement of health services delivery from the primary team in chronic diseases (either physician, nurse, nutritionist, or other) compared to the previous standard care. Of the surveyed patients, 71% considered an improvement, 25,7% considered no improvement, and 3,3% did not answer the question.

### Qualitative results

Core elements of the MPCM were identified in the patient’s answers. Case management had a higher association with the support on the coordination of appointments in secondary and tertiary care and with open communication, referring to the possibility of contacting and being contacted directly by the nurse or physician via text messaging or telephone call. Thus, they consider that this reciprocal communication implies “greater closeness” with their primary team and better access to care throughout the health system.

In addition, Continuity of Care, Participation, and Shared Responsibility had the same proportion of positive perceptions about the changes made. Support for Self-Management was the least mentioned. They perceive emerging information as important non-clinical aspects of the center, such as cleanliness, order, and comfort.

*“Because within the services of the MPCM the health team is in permanent contact, … they are always calling me when I am entitled to an exam. It is excellent that nurses are active and look after us …, always good care” (P98, Pos. 1)*.

The **most valuable core element** was the care they received from clinical consultants within the MPCM. Examples referring to *Case Management* and *Participation and Shared Responsibility* emerged first. An improved patient experience was identified as an emerging topic and a greater perception of solution.

*“Because one is heard … what we feel and what is our concern. For example, he prescribed me a new drug, eliminated my clonazepam…then he called me to ask me how I was feeling… When they ask me to have some clinical tests, he then calls me to ask me about the results. Thus, we need to go less to the PHC” (P53,2)*.

On the contrary, **the least valuable** core element of the MPCM was related to the continuity of care and structural aspects of the health system. For example, there are complications in scheduling an appointment by phone and the need for continuity of care with the primary physician.

The context of the pandemic emerges as a relevant aspect since they consider that it has implied changes in their healthcare teams and a decrease in access to certain healthcare professionals. COVID-19 prioritization and organization were considered barriers to receiving care in some cases. However, in other patients, the pandemic allowed new alternatives to health services delivery, such as home clinical care and delivery of drugs to their homes.

## Discussion

The evaluation of patients’ perspectives on implementing the MPCM complements previous evaluations on the impact on health services utilization of the clinical intervention for high-risk patients [25]. The study has shown positive results from the qualitative and quantitative high-risk patients’ perspectives regarding the core elements of the new model implemented in six primary care centers for adults with multimorbidity. The main findings conclude that there was a positive overall satisfaction with implementing the MPCM. Core elements such as case management had a positive patient perspective, followed by constant communication with the primary health care teams. On the contrary, the least valued element was the limited continuity of care.

The MPCM strongly focuses on individualized care plans to drive the change from a single diagnostic to a person-centered approach. During the study, personalized clinical consultants have been identified as a positive aspect, where case management, risk stratification, and shared responsibility were considered core elements and drivers of individualized services. Therefore, the changes implemented in clinical care are improving patients’ perceptions, providing further insights apart from clinical outcomes and economic evaluations. Patient perspective results of this study reveal from the final and main care consumer that the core elements of the MCPM can positively impact patients’ experience, serving as a referent for other countries in developing their multimorbidity approaches [2, 4, 29].

Moreover, case management scored the highest in the qualitative and quantitative surveys. Although this care is more intensive and therefore requires a higher compromise of the patient with the clinical team, it strengthens communication and access results in a positive patient experience. In other studies where case management has been implemented, positive results have been found, especially in health services utilization, but there is a lack of findings from patients’ perspectives [30–32]. This study certainly contributes to the emerging knowledge in case management and multimorbidity, providing insights for decision-makers and public policy to complement results in health system performance.

In contrast, the less valued core elements were the continuity of care and self-management. The MPCM implemented strategies to enhance both, like self-management workshops and constant communication between primary and secondary care. However, fragmentation is still embedded in the clinicians and the structure of the health system. Despite those other countries and global organizations taking action to address multimorbidity [33], the COVID-19 pandemic deepened the problem of prioritizing covid related diseases, fragmenting care, and interrupting continuity and access [34–36]. Some of the finding support that argument. Thus, it is important to mention that this study performed the survey during the first year of the pandemic, which could have potentially biased the patients’ perspective from the services received with the MPCM.

One of the strengths of this study is that patients surveyed where those who have frequent service utilization given their complex health conditions. Therefore, they could provide a more realistic perspective of the health services received than patients with less contact with the primary care centers. In addition, the telephone survey allowed us to reach a high number of patients, representative of the total intervened patients with the MPCM, facilitating access to those patients’ patients that were not receiving face-to-face consultations during that specific time in the center due to the COVID-19 pandemic. Therefore, savings were for the study researchers and patients who did not have to spend time and money traveling.

The study has some limitations. The questionnaire was not validated because of the lack of time and resources; however, it was revised by an expert team and tested. Other limitations were proper from the telephone survey [37]; nevertheless, it facilitated access, especially during a pandemic. Professionals received the necessary training to ask for the information and complement the impact evaluation performed in the same strategy. In addition, given that pandemic triggered a crucial emotional component in the population [38] more time was given to the interviewed patients that needed to respond appropriately to the survey.

This study provides essential information for an emerging topic around the globe, such as addressing multimorbidity. In addition, the value of patient perspective in complex implementation, for example, in the one that the MPCM pursues, could serve as a referent for other countries in Latin America starting the process. Therefore, measuring both health services impact and patients´ perspective provides a more comprehensive view of the results and, therefore, could be used by a wider variety of health decision-makers.

Further research could be performed to evaluate the perspective of moderate and low-risk patients to have a complete panorama of all chronic patients according to their risk. A natural next step will be to compare these results with those primary care centers that have not intervened with the MPCM to measure the differences and provide strategies to address the gaps or weaknesses found. This health services and research agenda is especially relevant in countries where there is an urgent need to the scale-up of the change from a single diagnostic to a person-centered approach is already taking place, where the patient’s perspective should be on the agenda of the decision-makers.

### Electronic supplementary material

Below is the link to the electronic supplementary material.

Supplementary Material 1

## Data Availability

All data generated or analyzed during this study are included in this published article and its supplementary information files.

## Abbreviations

ACG: Adjusted Clinical Groups
CISAUC: Centro de Innovacion en Salud ANCORA UC
CESFAM: Family Health Center
MPCM: Multimorbidity Patient-Centered Care Model
FONASA: National Found of Health
NCD: Non-communicable diseases
PHC: Primary health care centers
SSMSO: Servicio Metropolitano Sur Oriente
SPSS: Statistical Package for the Social Sciences
