# Supplementary material for: Evaluation of patients´ perspective on a multimorbidity patient-centered care model piloted in the chilean public health system
Source: BMC Public Health. 2023 Nov 16;23:2264. doi: 10.1186/s12889-023-17220-3 (PMC10655404; doi:10.1186/s12889-023-17220-3)
Supplement: Supplementary file 1 — Supplementary Material 1 [file 12889_2023_17220_MOESM1_ESM.docx]

Supplementary Material

Telephone-based survey

|  | *Where 1 is very dissatisfied and 7 is very satisfied | | | | | | |
| --- | --- | --- | --- | --- | --- | --- | --- |
| 1. I received personalized attention, which responds to my specific needs for the management of my diseases (personalized attention for high risk, stratification for risk) | 1 | 2 | 3 | 4 | 5 | 6 | 7 |
| 2. I am cared for by the same nurse, and is the one who follows up on my care regularly (case management) | 1 | 2 | 3 | 4 | 5 | 6 | 7 |
| 3. I feel that the nurse who sees me regularly is reliable, and I can contact her if I need it (case management) | 1 | 2 | 3 | 4 | 5 | 6 | 7 |
| 4. I believe that when services or appointments with specialists or exams have to be coordinated, I receive support from the nurse. | 1 | 2 | 3 | 4 | 5 | 6 | 7 |
| 5. I feel able to manage my symptoms because my health team (doctor, nurse, nutritionist) has worked with me skills for managing my illnesses (e.g. measuring my blood pressure) (self-management support) | 1 | 2 | 3 | 4 | 5 | 6 | 7 |
| 6. I believe that the care received responds to my health needs, so I go less to other services, such as emergencies or hospitalizations (self-management support) | 1 | 2 | 3 | 4 | 5 | 6 | 7 |
| 7. I believe that the different health professionals who attend me at CESFAM are coordinated and aware of my health situation (continuity of horizontal care) | 1 | 2 | 3 | 4 | 5 | 6 | 7 |
| 8. When I have had to go to hospital or specialties, my family nurse is aware of the information about it (they ask me by phone or when I go back to my PHC) (continuity of vertical care) | 1 | 2 | 3 | 4 | 5 | 6 | 7 |
| 9. The nurse or doctor who attends me regularly at PHC, explains the changes or advances and together we decide how we continue with the management of my diseases (participation and shared responsibility) | 1 | 2 | 3 | 4 | 5 | 6 | 7 |

| 10. Overall, how satisfied you are with the care you receive for the treatment of your chronic illnesses | Very dissatisfied |  |  |  |  |  | Very satisfied |
| --- | --- | --- | --- | --- | --- | --- | --- |

| 11. Have you noticed improvement in the services you receive from the family team (doctor, nurse, nutritionist or other)? wherein? | Yes (specify) | No (specify) | Not applicable (less than 3 years) |
| --- | --- | --- | --- |
| 12. What you liked or valued most about the care you receive for the care of your chronic illnesses and why |  | | |
| 13. What you liked least about the care she receives for her chronic illnesses and why |  | | |
